# Research: Treatment: Relative risk of acute pancreatitis in initiators of exenatide twice daily compared with other anti-diabetic medication: a follow-up study

**DOI:** 10.1111/j.1464-5491.2012.03652.x

**Published:** 2012-10-08

**Authors:** M Wenten, J A Gaebler, M Hussein, E M Pelletier, D B Smith, P Girase, R A Noel, D K Braun, G L Bloomgren

**Affiliations:** 1 Amylin Pharmaceuticals Inc.San Diego, CA; 2 IMS Health IncorporatedWatertown, MA; 3 Eli Lilly and Company, IndianapolisIN, USA

## Abstract

**Aims** Previously, a retrospective cohort study found no increased risk of acute pancreatitis with current or recent use of exenatide twice daily compared with use of other anti-diabetic drugs. This follow-up study investigated incident acute pancreatitis, with the use of a different data source and analytic method, in patients exposed to exenatide twice daily compared with patients exposed to other anti-diabetic medications.

**Methods** A large US health insurance claims database was used. Eligible patients had ≥months continuous enrollment without a claim for pancreatitis and a claim for a new anti-diabetic medication on or after 1 June 2005 to 31 March 2009. Cases of acute pancreatitis were defined as hospitalized patients with an Internation Classification of Disease9 code of 577.0 in the primary position. A discrete time survival model was used to evaluate the relationship between exenatide twice daily and acute pancreatitis.

**Results** Of 482034 eligible patients, 24237 initiated exenatide twice daily and 457797 initiated another anti-diabetic medication. Initiators of exenatide twice daily had more severe diabetes compared with initiators of other anti-diabetic medications. After adjustments for propensity score, insulin and use of medication potentially associated with acute pancreatitis, the odds ratio with exenatide twice daily exposure was 0.95 (95%CI 0.65–1.38). A secondary analysis that examined current, recent and past medication exposure found no increased risk of acute pancreatitis with exenatide twice daily, regardless of exposure category.

**Conclusion** This study indicates that exposure to exenatide twice daily was not associated with an increased risk of acute pancreatitis compared with exposure to other anti-diabetic medications. These results should be interpreted in light of potential residual confounding and unknown biases.

## Introduction

Patients with Type2 diabetes are at a two- to threefold greater risk of acute pancreatitis compared with patients without diabetes [1–3]. Some co-morbidities of Type2 diabetes have been associated directly or indirectly with pancreatitis; for example, gallstone disease is a potential risk factor for acute pancreatitis, whereas obesity is a risk factor for increased complications of pancreatitis [4–5].

While there has been speculation that incretins may play a role in pancreatic duct cell proliferation [7–9] and varying results have been reported for markers of pancreatitis in several animal models in which rodents were exposed to such agents [10–12], to date a biologic mechanism for incretin-induced pancreatitis has not been identified in humans. Similarly, no signal for pancreatitis arose in the exenatide [a glucagon-like peptide1 (GLP-1) receptor agonist] clinical development programme, or in non-clinical studies in mice, rats and dogs of up to 24months in duration [13,14]. Randomized, clinical trials with open-label extensions (up to 3years) have demonstrated exenatide twice daily to be generally safe, with mild-to-moderate nausea reported as the most common side effect [15].

Since 2005, over 1million patients worldwide have been exposed to exenatide twice daily, accumulating more than 750000 patient-years of marketed exposure [16]. Post-marketing reports of acute pancreatitis with exenatide twice daily use in some patients prompted a pharmacoepidemiology study to examine the potential for an association between exenatide twice daily and acute pancreatitis. In a retrospective cohort study that used claims from the National Health Informatics Database, multiple analyses revealed that exenatide twice daily use was not associated with an increased rate of acute pancreatitis compared with use of other anti-diabetic medications; however, results from one analysis revealed an increased rate of acute pancreatitis among past users of exenatide twice daily compared with users of other anti-diabetic medications [17].

The objective of this follow-up analysis was to evaluate the occurrence of incident acute pancreatitis with exposure to exenatide twice daily compared with exposure to other anti-diabetic medications; a distinct US healthcare database and a unique time-varying exposure design were used. It was hypothesized that the relative risk of newly diagnosed acute pancreatitis would not be increased for patients who were exposed to exenatide twice daily compared with those who were exposed to other anti-diabetic medications.

## Subjects and methods

### Study design

This retrospective cohort study used the IMS LifeLink− Program Health Plan Claims (US) Database (http://www.imshealth.com/portal/site/ims/menuitem.edb2b81823f67dab41d84b903208c22a/?vgnextoid=78ee3cf808882310VgnVCM100000ed152ca2RCRD). A time-varying exposure design was used to identify the first occurrence of acute pancreatitis [International Statistical Classification of Diseases and Related Health Problems, 9th revision (ICD-9) code 577.0 in the primary position of the claim] in hospitalized patients who were exposed to exenatide twice daily or to other anti-diabetic medications [i.e. metformin, sulphonylureas, thiazolidinedione, insulins, sitagliptin, pramlintide, non-sulphonylurea secretogogues (meglitinide analogues) or α-glucosidase inhibitors].

### Data source

The IMS LifeLink database comprises fully adjudicated medical and pharmaceutical claims for over 65million unique patient lives from 98 health plans across the USA. The database includes inpatient and outpatient diagnoses (ICD-9 format), procedure (Current Procedural Terminology, 4th edition and Healthcare Common Procedure Coding System formats), both retail and mail-order prescription records, available data on prescription claims that include the National Drug Code, day's supply and quantity dispensed. Allowed, paid and charged amounts are available for all services rendered, as well as dates of service for all claims. Other data available include patient demographics, product type, payer type, provider specialty and eligibility dates related to plan enrolment and participation.

The data were longitudinal and the average duration of enrolment was 2–3years. Patient data were de-identified in compliance with the Health Insurance Portability and Accountability Act; therefore, this study was exempt from Institutional Review Board review.

### Participants

Patients with a claim for a new anti-diabetic medication of interest, including exenatide twice daily, on or after 1 June 2005 (the first date exenatide twice daily was commercially available), were eligible for cohort entry (Table 1). Eligible patients were those who were continuously enrolled in their health plan for ≥9months at baseline (pre-exposure) without a prior claim for exenatide twice daily or another anti-diabetic medication during the baseline period; therefore, enrolment began on 1 September 2004. Patients aged 65years and older at the initiation of the medication of interest who were not enrolled in Medicare Risk were excluded. Patients who entered the study before 65years of age, but did not participate in a Medicare Risk plan, were included and censored at age 65years. Patients with a gap in enrolment during follow-up were included in the study and censored at the start of the data gap. Patients with missing demographic or enrolment values, an invalid day's supply, not continuously enrolled or with a previous claim for pancreatitis (chronic or acute) during the baseline period were excluded. Patients were followed from the first day of cohort entry until the first incidence of acute pancreatitis, disenrolment from the health plan, death or until the end of available data (March 2009; but for most patients 31 December 2008). Of note, a diagnosis of Type2 diabetes was not required for inclusion in the analysis; however, the medications evaluated in the analysis are typically used only for the treatment of Type 2 diabetes. Also, there could have been claims for anti-hyperglycaemic medications before the index claim, but not for the index medication.

**Table 1:** Patient selection and cohort formation

Selection criteria	Patients removed (*n*)	Patients remaining (*n*)
Enrolment period I June 2005–31 March 2009	-	~36million
≥1 anti-diabetic medication claim on or after 1 June 2005	-	1256370
Age 65 years at initiation and not enrolled in Medicare Risk	208197	1048173
Missing demographic/enrolment data or invalid day's supply	90523	957650
Prior claim for study medication	196976	760674
9months of continuous health plan enrolment	275669	485005
Prior claim for pancreatitis	2971	482034
Hierarchical medication selection: exenatide twice daily>sitagliptin>insulin glargine	Exenatide twice daily (*n*)	Other anti-diabetic medication* (*n*)
Initiators eligible for cohort enrolment	24237	457797

^*^Other anti-diabetic medications were metformin, sulphonylureas, thiazolidinedione, insulins, sitagliptin, pramlintide, non-sulphonylurea secretagogues (meglitinide analogues), and α-glucosidase inhibitors.

### Statistical methods

#### Propensity score development

Inverse weighted propensity scores were used to account for differences in baseline characteristics between cohorts [18]. Over 600 independent variables were included, such as demographic characteristics, historical clinical characteristics, and the top 100 diagnoses, procedures, medications and top physician specialties during the pre-exposure period. These variables were incorporated into a logistic regression model to generate propensity scores [19]. The development of the propensity score was an iterative process that included modelling all potential main effects and interactions [20]. The model with the highest c-statistic was selected to generate the weights [21]. Variables indicative of insulin use were not included in the propensity score model as insulin use was a covariate in the discrete time survival model. The variable in the model is a binary variable (on/off) that indicates whether the patient was on or off exenatide during a certain period. The same variable also provides a mechanism in the model to control for exposure time. This is carried out through the concept of person-year. For each subject, the time during which he or she was receiving the drug contributed to 'on drug' time, and the time during which he or she was not receiving the drug contributed to 'off drug' time.

#### Primary analysis

The association between exenatide twice daily and acute pancreatitis was modelled with a discrete time survival model, where the time to acute pancreatitis event was a dependent variable and time-varying exposure status was the independent variable. The final regression model included time-varying covariates (insulin and drugs potentially associated with pancreatitis) and the propensity score to weight each observation. For the unadjusted analysis, incidence rates of disease and 95% confidence intervals (CI) were calculated per 100000 person-years of exposure.

#### Exposure

Exposure status was determined by whether the patient continued or discontinued exenatide twice daily as of the end of each observed follow-up interval (31days). Exposure was determined by examining the proxy date of the elapsed day's supply with a 31-day grace period. Patients exposed to exenatide twice daily in previous intervals who discontinued were assigned as non-exposed in the appropriate interval. For the secondary time-on-drug analysis, exenatide twice daily exposure was assigned to current, recent or past-use categories on the basis of the time of the most recent dispensing of exenatide twice daily. Patients were assigned to current exposure to exenatide twice daily if, on the last date in the interval, 31days or fewer had lapsed since the end of days supplied. Recent exposure extended 60days beyond current exposure, after which patients were classified as past users of exenatide twice daily.

#### Outcome

The primary outcome was defined as the first occurrence of acute pancreatitis (ICD-9 577.0 in primary position of the claim) that required hospitalization. For the outcome variable, an indicator was used to specify if the patient experienced an acute pancreatitis event. Patients with acute pancreatitis events were included in the analysis up to the interval (31days) during which the event took place and were censored from subsequent intervals.

#### Predictors

A priori confounders such as age, gender and geographic location were identified empirically. Additional potential confounders were derived from diagnosis, procedure and medication codes associated with the claims data in the database; however, potential risk factors for pancreatitis (e.g. smoking, alcohol use, high BMI, cholelithiasis) were not completely captured in the claims data. The Charlson co-morbidity index was calculated for each patient on the basis of the medical and hospital claims that were identified in the pre-exposure period.

#### Sensitivity

Sensitivity analyses included a crude intent-to-treat analysis, a survival model that used time-varying covariates with full adjustment and propensity score stratification [22,23]. Data management and analyses were conducted with the use of Statistical Analysis Software (SAS), versions8.2 and 9.1 (SAS Institute, Cary, NC, USA).

## Results

Initially, 24237 patients entered the exenatide twice daily initiator cohort and, at the end of the study, 42802 were exposed overall. For patients treated with other anti-diabetic drugs of interest, the initial cohort had 457797 patients enrolled and 478151 patients at the end of the study. Overall, patients in the exenatide twice daily cohort were more likely to have claims for medications at baseline that were potentially associated with pancreatitis and for co-morbidities (e.g. obesity, hyperlipidaemia) than were those from the other anti-diabetic medication cohort (Table 2). The average follow-up time was 1.4years for the exenatide twice daily cohort and 1.3years for the other anti-diabetic drug cohort. Overall, there were 46 acute pancreatitis events in the exenatide twice daily cohort and 802 events in the comparator group (Table 3); analysis of these data revealed similar crude acute pancreatitis incidence rates per 1000 person-years for both cohorts (exenatide twice daily: 1.32; other anti-diabetic drugs: 1.33; *P*=0.9383).

**Table 2:** Select patient baseline demographic and clinical characteristics

Baseline characteristics	Exenatide twice daily (*n*=24237)	Other anti-diabetic drugs* (*n*=457797)
Age, years, mean (sd)	52 (9)	51 (14)
Age strata, %		
0–34years	5	12
35–44years	14	15
45–54years	35	29
55–64years	45	38
≥65years	1	6
Male/female, %	42/58	47/53
Geographic distribution, %		
North-east/mid-west/south/west	28/26/38/8	30/35/24/11
Select diagnoses, %		
Diabetic retinopathy	10	5
Peripheral neuropathy	5	3
Hyperlipidaemia	64	46
Hypertension	63	51
Obesity	16	9
Select medications, %		
Angiotensin-converting enzyme inhibitors	34	21
Angiotensin receptor blockers	16	8
ClassI pancreatoxic [30]	46	34
ClassII pancreatoxic [30]	51	39
Statins	47	28
Fibrates	12	6

*Other anti-diabetic medications were metformin, sulphonylureas, thiazolidinedione, insulins, sitagliptin, pramlintide, non-sulphonylurea secretagogues (meglitinide analogues) and α-glucosidase inhibitors.

**Table 3:** Intent-to-treat unadjusted acute pancreatitis rate

Acute pancreatitis events	Exenatide twice daily initiators (*n*=24237)	Other anti-diabetic drug initiators (*n*=457797)	*P*-value
Patients with event, *n*	46	802	
Person-years*	34958	602381	
Incidence rate/1000 person-years	1.32	1.33	0.9383
Rate ratio (95%CI)			
	0.99 (0.73–1.33)		

*Includes time from index date to first event or end of follow-up, whichever came first.

**FIGURE 1:**
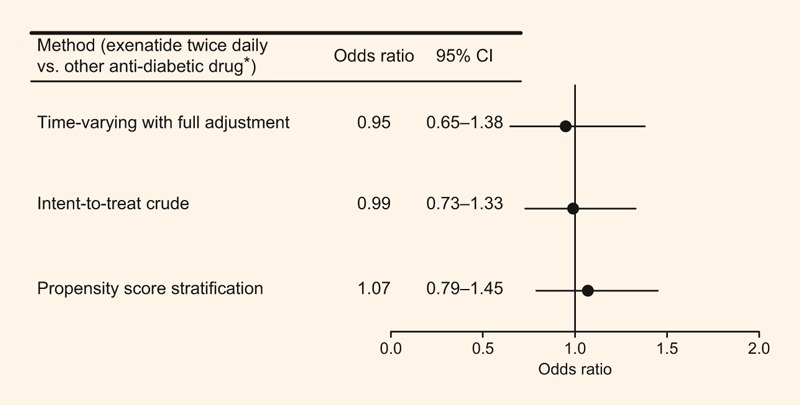
Risk of acute pancreatitis following exposure to exenatide twice daily. *Other anti-diabetic drugs were: metformin, sulphonylureas, thiazolidinediones, insulins, sitagliptin, pramlintide, non-sulphonylurea secretagogues (meglitinide analogues) and α-glucosidase inhibitors.

**FIGURE 2:**
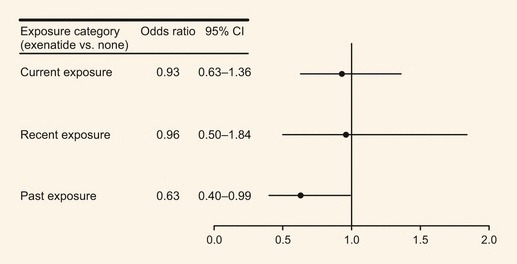
Risk of acute pancreatitis with exenatide twice daily by exposure category. *Current exposure, exenatide dispense date+days supplied+31days; recent exposure, end of current exposure+60days; past exposure, any days beyond end of recent exposure; none, no exenatide exposure.

In the primary analysis, exposure to exenatide twice daily was not associated with an increased risk of acute pancreatitis [odds ratio0.95 (95%CI 0.65–1.38), *P*=0.7772) compared with exposure to other anti-diabetic medications (Fig. 1). Point estimates for the sensitivity analyses ranged from 0.99 to 1.07 (Fig. 1). Results from the sensitivity analyses confirmed the results from the primary analysis, indicating that there is not an elevated risk of acute pancreatitis with the use of exenatide twice daily compared with the use of other anti-diabetic medications. Similarly, a secondary analysis that used a time-on-drug approach revealed that exposure to exenatide twice daily at any time (i.e. current, recent or past use) was not associated with an elevated risk of acute pancreatitis compared with exposure to other anti-diabetic drugs (Fig. 2).

## Discussion

Spontaneous reports of pancreatitis received with marketed use of exenatide twice daily have been suggestive of a potential association with acute pancreatitis. Known limitations to assessments of spontaneous reporting of safety signals include (1) variable reporting across products and time, (2) imprecise denominators attributable to uncertain exposure, (3) inconsistent report quality and (4) reporting biases that may be influenced by product approval date, nature of the adverse event and publicity. Results of the current analyses indicate that, compared with use of other anti-diabetic medications, use of exenatide twice daily was not associated with an increased risk of hospitalized acute pancreatitis. These results may be generalized to commercially insured patients with Type2 diabetes in the USA.

Epidemiologic studies such as the one at hand are prone to bias by indication. There are multiple statistical techniques that could be used to adjust for such biases, the simplest of which is matching patients on one or more key confounding variables. However, such simple techniques have the fundamental limitation of the inability of controlling for more than three confounding variables. The propensity score method allows the possibility for matching patients on many confounding variables. Propensity scores represent a patient's probability of receiving a given treatment option and are calculated by summing coefficient values for a list of potential confounding variables. The score is a summary of the likelihood (chance) of one patient being similar to another, conditional on an array of potential confounding variables.

The primary safety endpoint was the incidence rate of acute pancreatitis when the diagnosis of pancreatitis was in the primary position. However, we also evaluated these data when the diagnosis of pancreatitis was in the primary position or in any of the secondary positions. The results of this analysis showed a disproportionate increase in the captured event rates among the two exposure groups (27 vs. 39% for exposed and not exposed, respectively), indicating potential differential misclassification. Because of this, only diagnoses with pancreatitis in the first position were used.

In a previous time-on-drug analysis (i.e. the primary study), current and recent use of exenatide twice daily was not associated with an increased risk of acute pancreatitis compared with use of other anti-diabetic medications [17]. In that analysis, however, there was an observed increased risk of acute pancreatitis with past use of exenatide twice daily compared with use of other anti-diabetic medications; this effect was attenuated when covariates (e.g. obesity, gallstone disease and prior acute pancreatitis history), obtained by medical record abstraction, were included in the model. In order to determine reproducibility of the results of the primary study, the follow-up study, reported here, was designed with the use of a different model and a distinct claims database.

The main difference between the primary study and the follow-up study is that the primary study used medical record abstraction and adjudication to confirm claims-identified cases of acute pancreatitis. In the follow-up study, a claims-based definition of acute pancreatitis, rather than a clinical diagnosis of acute pancreatitis, was used. A discrete time survival model, which accounts for time-varying covariates, was used for the follow-up study to account for exposures that were related to both exenenatide twice daily exposure and acute pancreatitis. One feature of this model is that it accounts for switching, so patients who change medications also change cohorts over time; therefore, adverse events are attributed to patients' current therapy, rather than their initial therapy. An advantage to the inverse propensity scoring method used in this analysis is that it requires fewer assumptions about the underlying data than do methods that use propensity score matching or stratification. Inverse probability weighting avoids potential residual confounding of stratification on a fixed number of strata [23]. In addition, propensity score matching algorithms have limited generalizability attributable to omission of substantial unmatched portions of the population; however, this is not the case with inverse probability weighting.

Reports of the positive predictive value of claims-based definitions of acute pancreatitis vary from 42 to 82%, which may lead to misclassification bias [24–25]. The review of the medical records in the primary study found the positive predictive value to be 51% when the ICD-9 code (577.0) was in the primary position [27]. Misclassification would be expected to bias the relative risk toward the null, assuming that the classification errors are independent and non-differential across exposure categories. However, if prescribers preferentially use exenatide twice daily to treat their patients with more severe diabetes, there may be a bias away from the null. Adjustments for medication potentially associated with pancreatitis and insulin use resulted in a higher risk of acute pancreatitis, which may be an indicator of disease severity; however, further study is warranted to better understand this observation.

Recently, an observational study of pharmacy claims evaluated the risk of acute pancreatitis among patients with and without diabetes and among patients treated with exenatide twice daily, sitagliptin or control diabetes medications. In that analysis of 786656 patients, no increased risk of acute pancreatitis was evident with the use of exenatide twice daily [adjusted hazard ratio0.9 (95%CI 0.6–1.5)] or sitagliptin [adjusted hazard ratio1.0 (95%CI 0.7–1.3)] vs. the use of control diabetes medications [3]. In addition, the investigators observed an increased risk of acute pancreatitis in patients with diabetes compared with those without diabetes. These findings were consistent with those of previous studies that indicated no increased risk of acute pancreatitis with exenatide twice daily or sitagliptin treatment relative to other anti-diabetic medication treatment [28] and an increased risk of acute pancreatitis in patients with diabetes compared with patients without diabetes [1,2].

In conclusion, results of this study showed no increased risk of acute pancreatitis associated with exposure to exenatide twice daily compared with exposure to other anti-diabetic medications; these results are consistent with those from a previous epidemiologic study, which used a different methodology and a different database [17]. In contrast with the earlier epidemiologic study, the results from current study did not reveal an increased risk in past exenatide twice daily users compared with other anti-diabetic medication users. These results should be interpreted in light of study limitations, residual confounding and other unknown biases.

### Limitations

A limitation of the analysis presented here is that the definition for acute pancreatitis outcomes may not meet clinical criteria (i.e. misclassification), which would result in the inclusion of false positives that may obscure a potential association. Although we controlled for a large set of factors that potentially differed between the non-randomized comparison groups at baseline with the use of propensity score methodology, the incomplete capture of variables in the claims data (e.g. obesity, smoking, alcohol consumption, gallstone disease) is a limitation of the present analysis. Indeed, evaluation of the baseline characteristics of the study cohorts suggests that exenatide twice daily-treated patients had a higher prevalence of co-morbidities than did other patients treated with anti-diabetes medication. However, the direction of imbalance in these partially captured variables suggests that remaining (unmeasured) confounding would lead to a spuriously higher risk of acute pancreatitis among exenatide twice daily users, a finding that was not observed. The claims data set does not include uninsured patients, patients 65years of age and older and not covered by Medicare Risk, or those covered only by Medicare (PartD); the source population consisted primarily of commercially insured patients in the USA. Therefore, the results are most generalizable to similar commercially insured patients and may not be generalizable to other populations if they differ in age or in their accessibility to physician services or prescriptions. However, this issue of generalizability would be unlikely to bias the finding as the process of exclusion is independent from the factors under study.

Another limitation of this study was that consumption of the medication was not directly measured but was assumed on the basis of submitted claims; however, this approach has been shown to accurately derive medication exposure from pharmacy claims [29]. Additional data are needed because of the small number of acute pancreatitis outcomes and the relatively short time since approval of exenatide twice daily, as indicated by fewer initiators of exenatide twice daily compared with initiators of other anti-diabetic drugs.

### Funding sources

None.

### Competing interests

This work was funded by Amylin Pharmaceuticals Inc. and Eli Lilly and Co., which have global agreement to jointly develop and commercialize exenatide. At the time of this study, MW, JAG, and GLB were employees and stockholders of Amylin Pharmaceuticals Inc. RAN and DKB are employees and stockholders of Eli Lilly and Company. IMS Health has consultant agreements with Amylin Pharmaceuticals Inc., Eli Lilly and Co., Sanofi-Aventis and Pfizer Inc.
